# Supplementary material for: Perihematomal Edema and Clinical Outcome in Intracerebral Hemorrhage Related to Different Oral Anticoagulants
Source: J Clin Med. 2021 May 21;10(11):2234. doi: 10.3390/jcm10112234 (PMC8196746; doi:10.3390/jcm10112234)
Supplement: Supplementary file 1 [file jcm-10-02234-s001.zip › jcm-1189614-supplementary.pdf]

Supplemental Material

Supplementary Table 1: Univariate analysis of predictors of poor outcome (modified Rankin Scale 4-6) in patients with oral anticoagulation associated intracerebral hemorrhage (OAC-ICH) at discharge.

	**Poor outcome (mRS 4-6) in OAC-ICH**			
**Predictor**	**OR**	**95% CI**	**Regression-Coefficient**	***P*-Value**
**Age [years]**	0.999	0.967-1.032	-0.001	0.945
**Gender [ref: female]**	1.112	0.536-2.307	0.106	0.776
**Hypertension [ref: yes]**	0.587	0.191-1.802	-0.534	0.351
**Diabetes mellitus [ref: yes]**	0.70	0.296-1.653	-0.357	0.416
**Anticoagulation**	-	-	-	-
**Antiplatelet [ref: yes]**	1.397	0.450-4.341	0.334	0.563
**symptom onset to imaging [hours]**	0.992	0.977-1.006	-0.008	0.258
**GCS on admission**	0.694	0.568-0.848	-0.365	<0.0001
**NIHSS on admission**	1.125	1.048-1.208	0.118	0.036
**ICH Volume [mL]**	1.026	1.011-1.042	0.026	0.001
**PHE Volume [mL]**	1.018	1.002-1.034	0.018	0.024
**rPHE**	0.930	0.766-1.129	-0.072	0.465
**EED [cm]**	1.031	0.879-1.210	0.031	0.709
**IVH [ref: yes]**	3.353	1.502-7.484	1.21	0.003
**Location (ref: supratentorial)**	0.738	0.355-1.534	-0.304	0.415
**Craniectomy [ref: supratentorial]**	1.977	1.014-3.856	0.682	0.045

Legend: ICH indicates intracerebral haemorrhage; IVH, intraventricular, GCS, Glasgow Come Scale; EED, edema extension distance; OAC-ICH, oral anticoagulation associated intracerebral hemorrhage; PHE, perihematomal edema; ref, reference; and rPHE, relative perihematomal edema.

Supplementary Table 2: Univariate analysis of predictors of poor outcome (modified Rankin Scale 4-6) in patients with non-oral anticoagulation associated intracerebral hemorrhage (NON-OAC-ICH) at discharge.

**Predictor**	**Poor outcome (mRS 4-6) in NON-OAC-ICH**			
	**OR**	**95% CI**	**Regression-Coefficient**	***P*-Value**
**Age [years]**	1.007	0.994-1.021	0.007	0.276
**Gender [ref: female]**	0.906	0.603-1.361	-0.099	0.634
**Hypertension [ref: yes]**	0.843	0.531-1.339	-0.171	0.469
**Diabetes mellitus [ref: yes]**	1.346	0.711-2.547	0.297	0.361
**Anticoagulation**	-	-	-	-
**Antiplatelet [ref: yes]**	0.956	0.61-1.498	-0.045	0.843
**symptom onset to imaging [hours]**	1.0	0.999-1.002	0.000	0.697
**GCS on admission**	0.766	0.712-0-824	-0.267	<0.0001
**NIHSS on admission**	1.128	1.086-1-172	0.121	<0.0001
**ICH Volume [mL]**	1.024	1.016-1.033	0.024	<0.0001
**PHE Volume [mL]**	1.023	1.014-1.032	0.023	<0.0001
**rPHE**	0.942	0.874-1.016	-0.059	0.122
**EED [cm]**	1.115	1.024-1.213	1.09	0.012
**IVH [ref: yes]**	5.177	3.227-8.304	1.644	<0.0001
**Location (ref: supratentorial)**	0.551	0.357-0.851	-0.596	0.007
**Craniectomy [ref: supratentorial]**	2.877	1.433-5.779	1.057	0.003

Legend: ICH indicates intracerebral haemorrhage; IVH, intraventricular, GCS, Glasgow Come Scale; EED, edema extension distance; PHE, perihematomal edema; ref, reference; rPHE, relative perihematomal edema; and NON-OAC-ICH, non-oral anticoagulation related intracerebral hemorrhage.
